# Progress in Dietetics

**Published:** 1904-04-30

**Authors:** 


### PROGRESS IN DIETETICS.

The dietetic treatment of disease, though it has long been recognised in a vague way, has only recently begun to assume the importance of a branch of exact science. The same is true of the dietetics of health. Weber[1] has laid down certain rules for the physiological regulation of diet. Moderation in the most nourishing articles of diet is the first law of health. He considers that many people believe that abstinence from alcohol is an excuse for over-indulgence in such stimulating foods as meat or the pulses. Though admitting that great individual differences exist, he considers that minimal amounts of animal food are essential to the prolongation of life, especially in old people. Only 5 per cent. of those above 80 are large consumers of meat. This was observed so long ago as 1568 by Lornaro and was confirmed by the teaching of Abernethy. With regard to old people he observes that a gradual loss of weight is normal, and no attempt should be made to counteract it by increased ingestion of food. Thorough mastication and abstinence from large draughts of water during meals are also essential to health. Among the labouring classes it has been shown[2] that great ignorance exists as to the relative values of foodstuffs. To remedy the main faults, the following points are insisted on: (i.) a diet of tea and bread with or without butter is faulty; (ii.) this can be corrected by the free use of animal food, but only at great expense; (iii.) oatmeal, peas or beans will correct the fault much more cheaply; (iv.) better methods of cooking are essential where economy is an object. It is proved that the actual cost of a labouring man's diet is almost double what it should be. The cheapest energy-provider is sugar the cheapest proteid-provider, peas; oatmeal is the cheapest provider of both. Bread is also a cheap food, and so is cheese. Suet and dripping are a neglected and cheap source of energy. Beef, fish, (fresh or dried) and eggs are very expensive as sources both of proteid and energy. Though largely used, potatoes are about twice as expensive as bread, and milk is even dearer. The anti-scorbutic properties of both especially the former, are, however, valuable. The question of alcoholic beverages is closely allied to dietetics. Weber[3] controverts the popular idea that alcohol is essential to old people. It diminishes the resisting powers of the tissues to disease, and is especially injurious to those whose arteries are degenerating. Only a few hard drinkers are long-lived, and of 4,284 persons it was found that the majority of long-lived individuals were total abstainers. Moore,[4] from life-assurance statistics, has conclusively proved that abstainers show marked superiority over non-abstainers through the entire working years of life in every class and in both sexes. The effects of alcohol are especially seen in the case of mothers among the labouring classes[5] 56 per cent. of the offspring of inebriate women die at birth or under two years of age, while in the case of sober women only 26 per cent. die. The State has spent £39,000 on inebriate women, and 9·6 per cent. of insanity in women is due to alcohol. In this connection some interesting facts have been collected with regard to the consumption and manufacture of whisky[5], 35 million gallons of which have been consumed in the United Kingdom during the last five years. A great change has occurred in its mode of manufacture. Twenty years ago only 30 per cent. was distilled from maize, molasses, etc., and 70 per cent. from barley malt, whereas now the proportion is reversed. The main difference in the two liquors lies in the by-products which give character to the beverage produced. Possibly the most important of these are the aldehydes, but the whole question is so obscure that the Royal Commission which reported in 1891 refused to recommend legislation. It seems clear, however, that much of the patent-still or "grain" spirit is very imperfectly rectified, and is largely substituted in "blends" for the genuine malt whisky, especially for exportation to British colonists and to native races in the tropics.

[1] Brit. Med. Jour., Dec. 5, 1903, p. 1447. [2] Ibid., p. 1471. [3] Loc. Cit. [4] Ibid., Jan. 16, 1904, p. 155. [5] Ibid., Dec. 26, 1903. p. 1645.

